# Effects of bacteriophage cocktail supplementation in gestation diet on reproductive performance, blood profile, milk composition, and fecal microflora of sows

**DOI:** 10.5713/ab.24.0796

**Published:** 2025-02-27

**Authors:** Jinsu Hong, Geon Il Lee, Jae-Cheol Jang, Yoo Yong Kim

**Affiliations:** 1 Department of Agricultural Biotechnology, Center for Food and Bioconvergence, and Research Institute of Agriculture and Life Sciences, Seoul National University, Seoul, Korea; 2 Department of Animal Science, University of Minnesota, Saint Paul, MN, USA; 3 Department of Agricultural Science, Korean National Open University, Seoul, Korea; 4 Division of Animal Science, and Institute of Agricultural and Life Science, Gyeongsang National University, Jinju, Korea; 5 Institute of Green Bio Science and Technology, Seoul National University, Pyeongchang, Korea

**Keywords:** Bacteriophage, *Escherichia coli*, Gestating Sows, Lactobacillus, Salmonella

## Abstract

**Objective:**

The present study aimed to evaluate the effects of bacteriophage cocktail supplementation in diet of gestation sows on both the sows and their progeny.

**Methods:**

A total of 57 F1 multiparous sows (Yorkshire×Landrace) were allotted to one of three treatment groups in a completely randomized design. The sows were fed corn-soybean meal-based diet supplemented with 0%, 0.05%, or 0.10% of a bacteriophage cocktail during the gestation, followed by a common diet during lactation. Body weight and backfat thickness were measured during the trial along with blood collection for immunoglobulin analysis. Fecal samples were collected on 4, 6, 8, and 10 weeks of gestation period to examine fecal microflora. Litter performance and milk composition were investigated at 24 hrs postpartum and on d 21 of lactation.

**Results:**

Increasing bacteriophage cocktail supplementation levels in gestation diets resulted in linear increas (p<0.05) in feed intake of lactating sows and in the weight of live litters, while showing quadratic increas (p<0.05) in litter weight gain from d 0 to 21 of lactation. Dietary treatment had no impact on the serum concentrations of IgG and IgM in the sows. Increasing dietary bacteriophage cocktail levels in gestation diets resulted in a linear increas (p<0.05) in the fat content of colostrum. The elevation of dietary bacteriophage cocktail in microflora of sows resluted in a significant increase (p<0.05) in *Lactobacillus* count for d 63 and 105 of the gestation period, while concurrently decreasing *Escherichia coli* (*E. coli*; p<0.05) and *Salmonella* (p<0.10) counts during the late gestation period.

**Conclusion:**

The supplementation of a 0.05% bacteriophage cocktail in the diet of gestating sows could improve total litter weight, alive litter weight at birth, and litter weight gain during the lactation. This effect is attributed to positive changes in fecal microflora with an increase of *Lactobacillus* and a decrease of *E. coli* and *Salmonella*.

## INTRODUCTION

The prohibition of antibiotics as growth promoter for swine industry in United States and ban on routine antibiotic use including preventative group treatments in European Union has reflected growing concerns regarding antibiotic-resistant microorganisms [1]. Decreasing frequency of exposure to antibiotics, however, has been challenged due to the prevalence of enteric disease and poor gut health of pigs after weaning. These problematic circumstances with drug-resistant bacteria such as *Escherichia coli* (*E. coli*) and *Salmonella* spp. [2] can influence vertical transmission of the resistant bacteria from dam to her offspring, raising concerns about the persistence and propagation of resistance within swine population [3]. In pigs, previous findings provide evidence that alternatives for antibiotics such as prebiotics, probiotics, organic acids, plant extracts, or some components of dietary fiber have shown inconsistent effects with variable results in improving gut health and reducing symptom of pathogen infection [1,4–5]. Given these problems and findings, there is a critical need to characterize and develop feed additives as antibiotics alternatives, especially targeting the specific pathogenic bacteria residing in commercial pig barns.

Bacteriophages, or phages, are viruses that selectively target bacterial cells. Using their tail fibers, they can attach to bacterial surface and thereby injecting their genetic materials for bacteriophage replication into the bacterium [6], ultimately leading to the decrease in proliferation of the targeted bacterial population [7,8]. It implies that bacteriophage can kill the targeted bacteria such as *Salmonella* and *E. coli* without affecting other bacteria species, hence being used as an antibacterial agent [9]. Previous studies have demonstrated the antimicrobial efficacy of bacteriophages against *Salmonella* [10,11] and *E. coli* [12,13] in pigs. Supplemental effects of the bacteriophage cocktail for pigs could be likely differed by their growing phase, bacteriophage type for target virus, health status of pig herd, or environment hygiene. Kim et al [14] reported that supplementation of bacteriophages at 0.1%, targeting for *Salmonella, Staphylococcus aureus, E. coli*, and *Clostridium perfringens* positively affect average daily feed intake and average daily gain (ADG) of growing pigs (50 kg body weight [BW]) with an improvement in apparent total tract digestibility (ATTD) of DM, gross energy, and crude protein (CP). Lee et al [15] found that bacteriophages supplementation at 0.1% (same product used in Kim et al [14]) in weaning pigs’ diet improved ADG with improvement in ATTD of DM and CP. In contrast, Yan et al [16] observed that supplementation of bacteriophage targeting for *Salmonella* spp. did not improve the growth performance of growing pigs (29 kg BW). In this regard, the practical application of bacteriophages remains a subject of debate according to swine herd, farm condition, growing phase, and different components in feed.

While the effects of bacteriophage supplementation on growth performance, fecal score, and gut health were reported for weaning pigs [15,17–19] and growing pigs [14,16], research on bacteriophage supplementation for sows is scarce. An increase in gut microbial diversity induced by bacteriophages has also been reported (as reviewed by Shkoporov and Hill [20]). Additionally, significant correlations were found between litter size and the gut microbiota of sows, which were likely associated with changes in plasma biochemical parameters, inflammatory factors, and immunoglobulins [21]. The effects of bacteriophage supplementation in gestating sow diets on reproductive performance, fecal microflora, and growth of their progeny have not been reported. Thus, it was hypothesized that supplementing a bacteriophages cocktail in the diets for gestating sows would reduce the populations of target *E. coli* and *Salmonella* in the gestating sow’s gut microflora, thereby enhancing their reproductive performance and immune status, leading to improved growth of their progeny. This study aimed to evaluate the effects of bacteriophage cocktail supplementation levels in the gestation diet on reproductive performance, physiological responses, and fecal microflora of gestating sows, as well as their subsequent physiological responses, litter performance, and milk composition during lactation.

## MATERIALS AND METHODS

All experimental procedures involving animals were conducted in accordance with the Animal Experimental Guidelines provided by the Seoul National University Institutional Animal Care and Use Committee (SNU-231203-2).

### Bacteriophage

The bacteriophage product used in the current study was obtained from a commercial feed company (CTCBio Inc., Hwaseong, Korea) by mixing of excipients with lyophilized bacteriophage cocktail infecting *Salmonella* (*S. typhimurium, S. enteritidis, S. cholerasuis*, and *S. derby*), *Staphylococcus aureus, E. coli* (k88, k99, and f41) and *Clostridium perfringens* types A and C. These bacteriophages are isolated from water, soil, and farm waste samples, and their antibacterial activities were confirmed by a conventional plaque assay. The titer of each bacteriophage in the bacteriophage cocktail was 10^9^ plaque-forming units (pfu)/g bacteriophage cocktail.

### Animals

A total of 57 F1 multiparous sows (Yorkshire×Landrace; average parity 5.2) with average BW of 227.4±24.0 kg, were allotted to one of three treatments based on the BW, backfat thickness (BFT) at P_2_, and parity in a completely randomized design when sows reached on day 35 of gestation. All sows had undergone two artificial inseminations, and their pregnancy were checked on day 34 of gestation by an ultrasound scanner.

### Experimental diets

Three experimental diets were a corn-soybean meal-based basal diet with supplementation of 0, 0.05, and 0.10% bacteriophage cocktail. The experimental diets for gestating sows contained 3,265 kcal of metabolizable energy (ME)/kg, 12.9% CP, 0.74% total lysine, and 0.23% total methionine. The other nutrients of the experimental diets meet or exceed the nutrient requirements of NRC [22]. The diet formulation and chemical composition of the experimental diets were presented in Table 1. The diets were ground into 1 mm particles by a Wiley mill (Wiley Mill Intermediate; Thomas Scientific, Swedesboro, NJ, USA). The experimental diets were analyzed for DM (procedure 967.03; [23]), crude ash (procedure 923.03; [23]), and CP by using the Kjeldahl procedure with Kjeltec (Kjeltec 2200; Foss Tecator, Hilleroed, Denmark; procedure 981.10; [23]). The lactation diet was the same commercial feed (Daehan Feed, Incheon, Korea) for lactating sows.

### Animal management

Gestating sows in second parity were fed 2.2 kg/d of experimental diet and gestating sows in over third parity were fed 2.4 kg/d of experimental diets once a day (08:00). Feed was gradually reduced 0.2 kg/d for 5 days before the date of farrowing. After farrowing, the amount of feed increased gradually by 1 kg/d during the first 5 days postpartum (1 to 5 kg/d). After then, they were fed the lactation diet and water *ad libitum* until weaning.

Sows were housed in individual gestation stalls (2.20×0.64 m) where an automatic ventilation system regulated the indoor temperature (average 20°C) by an automatic ventilation system. On day 110 of gestation, the sows were washed their bodies, including breasts and vulva, and moved to farrowing pens (2.50×1.80 m). All sows did not treat a delivery inducer, and they were given assistance for dystocia as needed. The room temperature in the lactation barn was kept at 28±2°C, and the place under a heating lamp was kept at 32±2°C. Air conditioning in the lactating barn was regulated automatically by a ventilation/air-conditioner system. After weaning, the sows were moved to the breeding barn for the next estrus cycle.

After farrowing, piglets were cross-fostered within the treatment group within 12 hrs postpartum to balance the suckling intensity of sows and equalize the litter size. Tail docking, iron injection (Fe-dextran 150 ppm; Gleptosil, Alstoe, UK), and castration (for male piglets) were performed on all piglets at 3 days after birth. The piglets were provided with sow’s milk only throughout the entire lactation period, with no supplementation of milk replacer.

### Body weight, backfat thickness, lactation feed intake, and weaning to estrus interval

The BW and BFT of sows were recorded at days 35 and 110 of gestation, 24 hrs postpartum, and day 21 of lactation. The BFT was measured at the P_2_ position by using an ultrasound device (Lean Meter; Renco Corp., Minneapolis, MN, USA). The daily feed intake and wastage were recorded during lactation to assess the lactation feed intake. Weaning-to-estrus interval (WEI) was recorded by monitoring for the first standing estrus after weaning.

### Reproductive and litter performances

After the completion of farrowing, the number of piglets for total born, including stillbirth, mummy, and born alive, were recorded, and the individual piglet BW, including alive piglets, stillborn, and mummy were measured. When measuring the BW of piglets, ear notching was performed for the experiment and they were cross-fostered among the same treatment group within 12 hrs postpartum. The number of piglets and their BW were measured on day 21 of lactation to calculate litter weight, piglet weight, and their weight gain.

### Blood immune response

Blood collection for 4 sows per each treatment was taken by venipuncture of the jugular vein using 10 mL disposable syringes on days 35 and 110 of gestation, 24 hrs postpartum, and day 21 of lactation. The collected blood samples were transferred into serum tubes (SST^M^II Advance; BD Vacutainer, Becton Dickinson, Plymouth, UK) and centrifuged at 1,957×g and 4°C for 20 minutes (5810R; Eppendorf, Hamburg, Germany) after clotting at room temperature for 30 minutes. The serum was separated into a microtube and stored at −20°C in a freezer to determine immunoglobulins concentration. The serum concentration for immunoglobulins G and M (IgG and IgM) were analyzed using an immunoassay analyzer with nephelometry method (Dimension; Siemens, Washington, DC, USA).

### Milk composition

Colostrum and milk samples were taken from four sows per each treatment on 24 hours postpartum and day 21 of lactation. Colostrum and milk samples were collected from the first and second teats after an intravascular injection with 5 IU oxytocin (Komi oxytocin;, Komipharm International Co., Ltd., Siheung, Korea) into the ear vein. Collected milk samples were stored in a −20°C freezer until further analysis. The contents of casein, fat, protein, lactose, total solid, solid not fat, and free fatty acid in colostrum and milk samples were determined by using a Milkoscan FT 120 (FOSS, Hillerod, Denmark).

### Fecal microbial composition

Fecal *Lactobacillus, E. coli*, and *Salmonella* counts were measured at 4, 6, 8, and 10 weeks of the experimental gestation period (days 63, 77, 91, and 105 of gestation). Fresh fecal sample was collected directly from four sows per each treatment by rectal palpation. Samples were diluted with 1 g of collected feces and 9 mL of distilled water. After mixing the solution completely, they were diluted to 1/10^5^ concentration of the initial diluted solution. Each diluted solution was spread in the Petri dishes having MacConkey agar (BBL; BD, Franklin Lakes, NJ, USA), *Lactobacilli* MRS agar (Difco; BD), and *Salmonella Shigella* agar (BBL; BD) with Bacto agar (Bacto; BD), respectively. The agar plates with spreading the diluted solution were incubated at 37°C for 24 hours. After incubation, the number of fecal *Lactobacillus, E. coil*, and *Salmonella* was counted.

### Statistical analysis

All collected data were analyzed by least squares mean comparisons and were evaluated with the general linear model (GLM) procedure of SAS (SAS Institute, Cary, NC, USA). Orthogonal polynomial contrast was used to determine linear and quadratic response by increasing the bacteriophage supplementation level. The sow or litter were used as an experimental unit. The statistical difference was determined significant at p<0.05, and the tendency was also considered 0.05≤ p<0.10.

## RESUTLS

Bacteriophage cocktail supplementation did not affect BW and BFT on day 110 of gestation (Table 2). Also, there were no significant effects of dietary bacteriophage cocktail levels on BW gain and BF gain during the gestation period from day 35 to 110. Increasing the supplemental level of bacteriophage cocktail from 0 to 0.1% tended to linearly increase (Linear, p = 0.08) the sow’s BW on day 21 of lactation (Table 3). However, bacteriophage cocktail supplementation in gestation diet had no effect on BFT on day 21 of lactation, nor BW and BF losses during the lactation. Increasing the level of bacteriophage cocktail supplementation from 0 to 0.1% in gestation diets linearly increased (Linear, p<0.05) the lactation feed intake of sows (d 0 to 21 of lactation). In addition, sows fed the gestation diet with bacteriophage cocktail had a greater (p<0.05) lactation feed intake than those fed the gestation diet without bacteriophage cocktail. The WEI was not influenced by bacteriophage cocktail supplementation in gestation diets.

There were no effects of bacteriophage cocktail supplementation on the number of total born, stillborn, mummy, and born alive (Table 4). However, increasing the supplemental level of bacteriophage cocktail in the gestation diet linearly increase (Linear, p<0.05) the alive litter weight at birth and tended to increase (Linear, p = 0.07) the total litter weight at birth. The litter weight and individual piglet weight after fostering (24 hrs postpartum) were increased linearly (Linear, p<0.05) as the bacteriophage cocktail supplementation level increased (Table 5).

Bacteriophage cocktail supplementation quadratically influenced (p<0.05) litter weight on day 21 of lactation and litter weight gain from day 0 to 21, such that both litter weight and litter weight gain increased (p<0.05) when dietary bacteriophage cocktail increased from 0% to 0.05% in the gestation period, and then decreased (p<0.05) when dietary bacteriophage cocktail increased from 0.05 to 0.1% in the gestation period. Similarly, piglet weight on day 21 of lactation tended to be quadratically increased (Quadratic, p = 0.05) and piglet weight gain was quadratically increased (Quadratic, p<0.05) by the increasing levels of dietary bacteriophage cocktail in gestating sows.

Bacteriophage cocktail supplementation did not affect the serum concentrations of IgG and IgM in sows on day 110 of gestation, 24 hrs postpartum, and d 21 of lactation (Table 6). Increasing dietary supplementation of bacteriophage cocktail from 0 to 0.1% in gestation diets linearly increased (Linear, p<0.05) the fat content in colostrum (Table 7), whereas it did not affect other components in colostrum. The contents of casein, protein, lactose, total solid, solid not fat, and free fatty acid in colostrum and milk on d 21 of lactation were not influenced by dietary bacteriophage cocktail levels provided to gestating sows (Table 7).

Increasing the supplementation level of dietary bacteriophage cocktail from 0 to 0.1% linearly increased (Linear, p<0.05) the counts of *Lactobacillus* on days 63 and 91 of gestation, while it linearly decreased (Linear, p<0.05) the counts of *E. coli* on d 105 of gestation and tended to decrease (Linear, p = 0.09) the counts of *E. coli* on day 91 of gestation in the feces of gestating sows. Besides, increasing the bacteriophage cocktail supplementation level from 0% to 0.1% tended to linearly decrease (Linear, p<0.10) the counts of *Salmonella* on days 77, 91, and 105 of gestation (Table 8).

## DISCUSSION

Regarding the results of the previous studies [14–17], dietary bacteriophage improved the nutrient utilization in pigs, resulted in the improved growth performance of the pigs in weaning or growing phases. In the current study, however, no differences in BW and BFT changes of sows during the gestation and lactation periods were observed, probably due to the physiological characteristics of the pregnant sows. The sows used in the current study were an average of 5.2 parity, and their body growth and digestive system development were fairly different from weaning pigs or growing pigs. Gestating sows beyond third parity have completed body growth, while weaning and growing pigs are still in the stage of body growth and organ development. Additionally, gestating sows prioritize the utilization of dietary nutrients for fetal development and growth. In this study, palm kernel meal (PKM) and wheat bran (WB) were utilized as dietary fiber sources to alleviate constipation in pregnant sows. The incorporation of these fiber-rich ingredients (PKM, 60% of total dietary fiber; WB, 45% of total dietary fiber) aims to promote gastrointestinal health and improve the overall well-being of sows by enhancing bowel movement and reducing the risk of the propagation of harmful bacteria [24,25]. Dietary fiber inclusion in the diet can significantly affect the composition and activity of gut microbiota, influencing overall gastrointestinal tract (GIT) function [26]. As increasing sows age, they develop an improved ability to degrade fiber fractions in the large intestine compared to weaning and growing pigs, due to increased colonization of carbohydrate-degrading microbiota [27]. This ability allows sows to utilize fiber more efficiently, promoting the proliferation of beneficial bacteria in the GIT, which may mask the effects of bacteriophage on BW and BFT, even though bacteriophage could enhance gut health in sows.

Improved fecal microbial composition with increased *Lactobacillus* population and decreased *E. coli* and *Salmonella* populations in the gestating sows fed the bacteriophage cocktail-supplemented diet could be another potential factor of positively affecting the improved total litter weight and alive litter weight observed in the current study. *Lactobacillus* spp. induce higher resistance to infection and gastrointestinal diseases and improve gastrointestinal function for pig health and performance [28]. Anti-pathogenic effects and anti-oxidative stress over time were evident [29–31], supporting the notion that *Lactobacillus spp*. leads to an increase in the beneficial gut microbial population through suppressing the pathogenic bacteria colonization, including *Salmonella, Clostridia*, and *Enterobacteria* by rapid utilization of energy source [32,33]. Furthermore, *Lactobacillus spp*. can generate short-chain fatty acids (SCFA) with the fermentation of carbohydrate substrate, which could be utilized for protein synthesis and energy metabolism in pigs [34,35]. Besides, Lactobacillus has shown positive correlations with BW gain and feed efficiency, which could be partly attributed to increased intestinal SCFA production and reduced inflammatory response [36]. Therefore, the positive changes in fecal microbial composition due to bacteriophage cocktail supplementation during the gestation period may partly contribute to improved sow gut health, leading to enhanced energy utilization, as evidenced by the increase in litter weight at birth. However, further research is needed to explore whether SCFA produced from gut microbial fermentation, stimulated by bacteriophage cocktail supplementation, improves energy efficiency to support fetal growth and development.

A bacteriophage is a kind of virus that attach to only bacteria cells with their tail fibers and inject the required amount of components for bacteriophage replication into the bacterium [6]. Virulent phages, which undergo the lytic cycle, replicate themselves using the host machinery and lyse the host cell, resulted in the cytolysis of the specific bacteria [7–9]. Previous studies have reported that bacteriophages supplementation reduced the populations of *Salmonella* and increased the populations of *Lactobacillus* and *Bifidobacterium* in the fecal microflora of the growing pigs [14,32]. In addition, Smith and Huggins [37] observed that a two-bacteriophage mixture showed the antimicrobial ability targeted against enterotoxigenic *E. coli* strain P433 in neonatal pigs. Also, Endersen et al [9] reported that bacteriophage lysed the *E. coli* strains in an in-vitro study and significantly reduced the diarrhea of weaning pigs infected with *E. coli*. Additionally, the supplementation of bacteriophages containing *Salmonella gallinarum*, *Salmonella typhimurium*, and *S. Enteritidis* at 0.05% increased *Lactobacillus* and decreased *E. coli* and *Salmonella* in the fecal microflora of growing pigs [16]. These are in line with the results of Lee et al [15] who found that bacteriophage-cocktail supplementation at 0.1% in lactation diet decreased the *Clostridium spp*. population for d 14 of lactation and *Coliforms* population for d 21 of lactation in the fecal microflora of lactating sows. Regarding the results of the previous bacteriophage in pig studies, the significantly increased populations of *Lactobacillus* and reduced populations of *E. coli* and *Salmonella* in the current study could partly have been due to the antibacterial effects of dietary bacteriophages, which had an activity of lysing *Salmonella* and *E. coli*. Furthermore, the positive changes in the gut microflora of gestating sows with increased *Lactobacillus* and decreased *E. coli* and *Salmonella* are believed to positively contribute to the improvement in sow’s health and litter performance.

It is well-established that a significant portion of dietary protein and energy intake is allocated toward fetal growth and reproductive organ development [22]. The current study observed a linear improvement in total litter weight and alive litter weight. In addition, the quadratic effects of bacteriophage cocktail supplementation in gestation diet were observed in the litter weight gain and piglet weight gain during lactation period. Unfortunately, we didn’t measure nutrient digestibility of the gestating and lactating sows, which is a critical limitation of this experiment; This study was designed to assess the effects of bacteriophage cocktail on microbial composition of the gestating sows and their reproductive performance. Therefore, further study is needed to investigate how changes in the gut microbial composition of gestating sows fed bacteriophage cocktail impact the nutrient digestibility in both gestating and lactating sows. In the current study, sows fed the gestation diets with bacteriophage cocktail had a greater feed intake for lactation period compared with those fed the gestation diet without bacteriophage cocktail. A greater feed intake of sows during the lactation period is important for improving milk production and litter growth [38]. Moreover, the initial composition of the intestinal microbiome in piglets can be significantly influenced by the microbial community established in sows. As previously discussed, sows that were administered a bacteriophage cocktail exhibited an increased abundance of *Lactobacillus* spp. This improvement in beneficial bacterial compositions may positively affect the intestinal health of the piglets, contributing to improved robustness and overall health outcomes. However, it should be noted that litter weight gain and piglet weight gain were quadratically decreased to levels comparable to the Control treatment as dietary bacteriophage cocktail levels increased from 0.05% to 0.1%, despite no difference observed in lactation feed intake of the sows. This suggests that the transfer of beneficial microbes from the sow to the piglet and high lactation feed intake of sows fed bacteriophage cocktail might be crucial factors in improving litter and piglet performance after birth. Nevertheless, the specific causality behind the quadratic response in litter weight gain at the higher level of 0.1% bacteriophage cocktail could not be determined within the scope of the current study.

The linear increase in colostrum fat content observed in the current study may be due to the increased SCFA production by gut fermentation with a change in gut microbial composition. The change in gut microbiome can influence microbial fermentation and SCFA production in the hindgut of pigs [39]. The produced SCFA by microbial fermentation can be used for de novo synthesis of milk fat in the mammary gland of sows [40]. As previously mentioned, *Lactobacillus* is beneficial bacteria contributing to positive interaction with host-microbiota, additional energy metabolism by SCFA production, and upregulation of the immune system [30,36]. In the current study, only changes in *Lactobacillus, E. coli*, and *Salmonella* population in feces were investigated, but these changes in the gut microflora during the gestation could potentially contribute to the colonization and interaction of other beneficial bacteria, resulting in the additional SCFA production and absorption for greater fat content of the colostrum.

In the current study, there were no effects of bacteriophage cocktail supplementation on the blood concentration of IgG and IgM in sows. Kim et al [14] reported that bacteriophage cocktail supplementation in diets from 0 to 0.15% for growing pigs had no significant influence on serum IgG, IgA, and IgM. Hosseindoust et al [19] also observed that bacteriophages supplementation at 0.1% in the weaning pigs’ diet did not affect the serum concentrations of IgG, IgA, and IgM in weaning pigs for d 7, 21, and 35. Chen et al [21] found that high-reproductive performance sow group showed greater plasma concentrations of TNF-α and IgM on day 100 of gestation. However, supplementation of bacteriophage cocktail in gestation diets didn’t significantly affect the blood IgG and IgM in gestating and lactating sows in agreement with the results of previous studies. A possible explanation for this observation is that the study was conducted in a sanitary, highly biosecured sow facility, where the sows were not exposed to disease. Also, while the dietary bacteriophage cocktail affected the fecal microbial composition of the gestating sows, it appears that it was not enough to impact blood immunoglobulins levels.

## CONCLUSION

In conclusion, supplementation of bacteriophage cocktail at 0.05% in the gestation sow’s diet resulted in enhanced total litter weight, alive litter weight, and increased litter weight gain during lactation through positive alternations in fecal microflora, characterized by increased numbers of *Lactobacillus* and decreased numbers of *E. coli*, and *Salmonella*, contributing to improved colostrum fat content and greater feed intake for lactation period.

## Figures and Tables

**Table 1:** The formulation and chemical composition of experimental diets

Item	Gestation diets^1)^		

	CON	B5	B10
Ingredients (%)			
Corn	73.52	73.47	73.42
Soybean meal	14.89	14.89	14.89
Wheat bran	2.33	2.33	2.33
Palm kernel meal	2.22	2.22	2.22
Tallow	3.00	3.00	3.00
L-lysine HCl (78%)	0.18	0.18	0.18
DL-methionine (99%)	0.03	0.03	0.03
MDCP	1.90	1.90	1.90
Limestone	1.33	1.33	1.33
Vit. mix^2)^	0.10	0.10	0.10
Min. mix^3)^	0.10	0.10	0.10
Choline chloride-50	0.10	0.10	0.10
Salt	0.30	0.30	0.30
Bacteriophage cocktail^4)^	0.00	0.05	0.10
Chemical composition			
Metabolizable energy^5)^ (kcal/kg)	3,265	3,265	3,265
Moisture^6)^ (%)	11.77	11.33	10.90
Crude ash^6)^ (%)	3.36	3.39	3.40
Crude protein^6)^ (%)	13.00	13.17	12.92
Ether extract^6)^ (%)	5.02	5.12	5.10
Crude fiber^5)^ (%)	2.75	2.75	2.75
Total lysine^5)^ (%)	0.74	0.74	0.74
Total methionine^5)^ (%)	0.24	0.23	0.23
Total methionine+cysteine^5)^ (%)	0.53	0.53	0.53
Calcium^5)^ (%)	0.90	0.90	0.90
Total phosphorus^5)^ (%)	0.70	0.70	0.70

1) CON, corn-SBM based diet; B5, basal diet with bacteriophage 0.05%; B10, basal diet with bacteriophage 0.10%.

2) Provided per kg of diet: Vitamin A, 8,000 IU; Vitamin D3, 1,600 IU; Vitamin E, 32 IU; d-biotin, 64 g; Riboflavin, 3.2 mg; Calcium pantothenic acid, 8 mg; Niacin, 16 mg; Vitamin B_12_, 12μg; vitamin K, 2.4 mg.

3) Provided per kg of diet: Se, 0.1 mg; I, 0.3 mg; Mn, 24.8 mg; CuSO_4_, 54.1 mg; Fe, 127.3 mg; Zn, 84.7 mg; Co, 0.3 mg.

4) The bacteriophage was provided by CTCBio Inc. (Hwaseong, Korea).

5) Calculated value.

6) Analyzed value.

**Table 2:** Effects of bacteriophage cocktail supplementation in the gestation diet on body weight and backfat thickness in gestating sows

	Treatment^1)^			SEM	p-value		
	
	CON	B5	B10		Trt	Lin	Quad
No. of sows (head)^2)^	19	19	19				
Body weight (kg)							
Day 35	225.6	230.1	225.6	3.62	0.84	0.99	0.56
Day 110	252.3	265.5	263.2	3.15	0.18	0.13	0.23
BW gain (35–110 d)	26.7	35.4	37.6	1.46	0.44	0.25	0.55
Backfat thickness (mm)							
Day 35	19.6	19.0	18.7	0.69	0.86	0.60	0.90
Day 110	23.6	23.4	22.7	0.86	0.94	0.76	0.84
BF gain (35–110 d)	4.0	4.4	4.0	0.42	0.88	0.69	0.75

1) CON, corn-SBM based diet; B5, basal diet with bacteriophage cocktail 0.05%; B10, basal diet with bacteriophage cocktail 0.10%.

2) Average parity of sows for CON, B5, and B10 treatments were 5.2, 5.2, and 5.3.

SEM, standard error of the mean; Trt, treatment; Lin, linear effect; Quad, quadratic effect.

**Table 3:** Effects of bacteriophage cocktail supplementation in the gestation diet on body weight and backfat thickness, feed intake, and WEI in lactating sows

	Treatment^1)^			SEM	p-value		
	
	CON	B5	B10		Trt	Lin	Quad
Body weight (kg)							
24 h postpartum	224.3	237.8	239.2	3.90	0.27	0.14	0.47
Day 21 of lactation	211.7	226.3	227.8	4.32	0.17	0.08	0.55
BW loss (0–21 d)	−12.6	−11.5	−11.4	1.91	0.55	0.29	0.86
Backfat thickness (mm)							
24 h postpartum	22.2	22.8	21.4	0.80	0.97	0.95	0.81
Day 21 of lactation	20.0	20.7	19.8	0.81	0.97	0.97	0.80
BF loss (0–21 d)	−2.2	−2.1	−1.6	0.43	0.99	0.93	0.96
Lactation feed intake (kg/d)	4.13^b^	5.52^a^	5.32^a^	0.209	0.03	0.02	0.13
WEI (d)	5.00	4.93	5.23	0.127	0.60	0.79	0.35

1) CON, corn-SBM based diet; B5, basal diet with bacteriophage cocktail 0.05%; B10, basal diet with bacteriophage cocktail 0.10%.

a,b Means in the same row with different superscript letters were significantly different (p<0.05).

SEM, standard error of the mean; Trt, treatment; Lin, linear effect; Quad, quadratic effect; WEI, weaning to estrus interval.

**Table 4:** Effects of bacteriophage cocktail supplementation in the gestation diet on sow reproductive performance

	Treatment^1)^			SEM	p-value		
	
	CON	B5	B10		Trt	Lin	Quad
No. of pigs (head)							
Total born	12.8	14.1	12.7	0.41	0.38	0.96	0.17
Stillborn	1.5	1.4	0.9	0.22	0.62	0.37	0.76
Mummy	0.4	0.3	0.2	0.09	0.72	0.43	0.94
Born alive	10.9	12.4	11.6	0.39	0.41	0.48	0.24
Total litter weight (kg)	17.68	19.53	20.20	0.571	0.19	0.07	0.67
Alive litter weight (kg)	15.47	17.38	18.70	0.570	0.09	0.03	0.82

1) CON, corn-SBM based diet; B5, basal diet with bacteriophage cocktail 0.05%; B10, basal diet with bacteriophage cocktail 0.10%.

SEM, standard error of the mean; Trt, treatment; Lin, linear effect; Quad, quadratic effect.

**Table 5:** Effects of bacteriophage cocktail supplementation in the gestation diet on litter performance during lactation period (0 to 21 d)

	Treatment^1)^			SEM	p-value		
	
	CON	B5	B10		Trt	Lin	Quad
No. of piglets (head)							
After-fostering	11.08	11.31	11.25	0.154	0.70	0.43	0.78
Day 21 of lactation	9.83	10.15	10.06	0.219	0.38	0.20	0.56
Mortality	1.25	1.15	1.19	0.182	0.82	0.59	0.75
Litter weight (kg)							
After-fostering	16.24^b^	16.75^ab^	18.28^a^	0.357	0.02	<0.01	0.34
Day 21 of lactation	47.07^b^	55.04^a^	48.52^b^	1.614	0.04	0.22	0.02
Weight gain (0–21 d)	30.83^b^	38.29^a^	30.24^b^	1.524	0.04	0.62	0.01
Piglet weight (kg)							
After-fostering	1.47	1.49	1.63	0.030	0.05	0.03	0.30
Day 21 of lactation	4.82	5.50	4.79	0.138	0.15	0.97	0.05
Weight gain (0–21 d)	3.35	4.01	3.16	0.133	0.08	0.66	0.03

1) CON, corn-SBM based diet; B5, basal diet with bacteriophage cocktail 0.05%; B10, basal diet with bacteriophage cocktail 0.10%.

a,b Means in the same row with different superscript letters were significantly different (p<0.05).

SEM, standard error of the mean; Trt, treatment; Lin, linear effect; Quad, quadratic effect.

**Table 6:** Effects of bacteriophage cocktail supplementation in the gestation diet on blood IgG and IgM levels in sows

Item (mg/dL)	Treatment^1)^			SEM	p-value		
	
	CON	B5	B10		Trt	Lin	Quad
IgG							
Day 110	548.7	461.0	494.0	21.05	0.29	0.31	0.22
24 h postpartum	495.5	647.0	498.5	83.65	0.79	0.99	0.51
Day 21 of lactation	791.0	710.5	752.7	35.88	0.71	0.70	0.48
IgM							
Day 110	115.3	145.3	136.0	6.55	0.22	0.22	0.19
24 h postpartum	130.3	89.3	128.0	9.55	0.24	0.93	0.10
Day 21 of lactation	99.5	76.1	108.6	11.24	0.46	0.73	0.25

1) CON, corn-SBM based diet; B5, basal diet with bacteriophage cocktail 0.05%; B10, basal diet with bacteriophage cocktail 0.10%.

SEM, standard error of the meanTrt, treatment; Lin, linear effect; Quad, quadratic effect; IgG, immunoglobulin G; IgM, immunoglobulin M.

**Table 7:** Effects of bacteriophage cocktail supplementation in the gestation diet on colostrum and milk (d 21 of lactation) compositions

Item (%)	Treatment^1)^			SEM	p-value		
	
	CON	B5	B10		Trt	Lin	Quad
Casein							
24 h postpartum	7.72	7.17	8.08	0.530	0.87	0.84	0.64
Day 21 of lactation	4.46	4.49	4.39	0.055	0.42	0.62	0.62
Fat							
24 h postpartum	5.81	6.97	7.26	0.436	0.08	0.04	0.39
Day 21 of lactation	6.47	6.05	6.43	0.237	0.81	0.96	0.53
Protein							
24 h postpartum	10.21	9.30	10.61	0.755	0.86	0.86	0.99
Day 21 of lactation	4.81	4.74	4.69	0.082	0.85	0.58	0.98
Lactose							
24 h postpartum	3.67	3.89	3.52	0.149	0.72	0.75	0.47
Day 21 of lactation	5.87	6.20	6.02	0.104	0.56	0.62	0.35
Total solid							
24 h postpartum	22.42	22.72	24.37	0.953	0.73	0.48	0.77
Day 21 of lactation	18.35	18.14	18.20	0.272	0.96	0.85	0.85
Solid not fat							
24 h postpartum	14.34	13.63	14.66	0.618	0.87	0.88	0.63
Day 21 of lactation	11.07	11.28	10.98	0.089	0.49	0.71	0.27
Free fatty acid							
24 h postpartum	2.93	3.72	3.61	0.229	0.38	0.28	0.39
Day 21 of lactation	4.50	4.70	4.39	0.212	0.86	0.86	0.61

1) CON, corn-SBM based diet; B5, basal diet with bacteriophage cocktail 0.05%; B10, basal diet with bacteriophage cocktail 0.10%.

SEM, standard error of the mean; Trt, treatment; Lin, linear effect; Quad, quadratic effect.

**Table 8:** Effects of bacteriophage cocktail supplementation in the gestation diet on fecal microflora in gestating sows

Item (log_10_CFU/mL)	Treatment^1)^			SEM	p-value		
	
	CON	B5	B10		Trt	Lin	Quad
*Lactobacillus*							
Day 63	3.32^b^	3.98^a^	4.30^a^	0.166	0.03	0.01	0.54
Day 77	4.75	4.93	5.17	0.112	0.33	0.15	0.90
Day 91	5.11^b^	5.19^b^	5.48^a^	0.069	0.03	0.01	0.35
Day 105	5.30	6.08	6.16	0.219	0.22	0.12	0.44
*E. coli*							
Day 63	4.04	3.05	3.17	0.531	0.75	0.54	0.68
Day 77	4.17	3.45	3.04	0.358	0.47	0.24	0.85
Day 91	5.05	4.59	4.28	0.183	0.23	0.09	0.83
Day 105	5.30^a^	5.01^a^	3.17^b^	0.140	0.03	0.02	0.22
*Salmonella*							
Day 63	3.76	3.00	3.24	0.203	0.31	0.31	0.29
Day 77	3.48	2.08	1.62	0.440	0.21	0.09	0.60
Day 91	4.60	4.49	3.72	0.216	0.19	0.09	0.47
Day 105	4.16	4.10	3.69	0.102	0.11	0.06	0.35

1) CON, corn-SBM based diet; B5, basal diet with bacteriophage cocktail 0.05%; B10, basal diet with bacteriophage cocktail 0.10%.

a,b Means in the same row with different superscript letters were significantly different (p<0.05).

SEM, standard error of the mean; Trt, treatment; Lin, linear effect; Quad, quadratic effect.
